# Achievement of appropriate cesarean rates using Robson’s 10-Group classification system in Brazilian private practice

**DOI:** 10.1186/s12884-023-05803-2

**Published:** 2023-07-10

**Authors:** Andrea Silveira de Queiroz Campos, Daphne Rattner, Carmen Simone Grilo Diniz

**Affiliations:** 1 Faculdade de Saúde Pública, Universidade de São Paulo, Av. Doutor Arnaldo, 715, 2º andar, Cerqueira César, São Paulo, 01246904 SP Brasil; 2 Faculdade de Ciências da Saúde, Universidade de Brasília, Campus Universitário Darcy Ribeiro, Asa Norte, Brasília, 70910900 DF Brasil

**Keywords:** Cesarean section rates, Supplementary health, Health Care Models, Patient-centered care, evidence-based practice, Humanized childbirth, Robson classification.

## Abstract

**Background:**

Increasing cesarean section (CS) rates are a global concern because they are related to higher maternal and neonatal complication rates and do not provide positive childbirth experiences. In 2019, Brazil ranked second globally, given its overall CS rate of 57%. According to the World Health Organization (WHO), populational CS rates of 10–15% are associated with decreased maternal, neonatal, and infant mortality rates. This study aimed to investigate whether multidisciplinary care following evidence-based protocols associated with a high motivation of both women and professionals for a vaginal birth leads to less overuse of CS in a Brazilian private practice (PP).

**Methods:**

This cross-sectional study evaluated CS rates by Robson group for women who sought vaginal birth in a private practice in Brazil comparing with Swedish data. Collaborative care of midwives and obstetricians who adopted evidence-based guidelines was offered. CS rates, overall and by Robson group, contribution of each Robson group to the overall CS rate, clinical and nonclinical interventions, vaginal birth, pre-labor CS, and intrapartum CS proportions were estimated. The expected CS rate was calculated using the World Health Organization C-model tool. The analysis used Microsoft Excel and R Studio (version 1.2.1335. 2009–2019).

**Results:**

The PP overall CS rate was 15.1% (95%CI, 13.4–17.1%) versus the 19.8% (95%CI, 14.8–24.7%) rate expected by the WHO C-model tool. The population included 43.7% women in Robson Group 1 (nulliparous, single, cephalic, at term, spontaneous labor), 11.4% in Group 2 (nulliparous, single, cephalic, at term, induced labor or CS before labor), and 14.9% in Group 5 (multiparous women with previous CS), the greatest contributors to higher CS rates (75.4% of them). The Swedish overall CS rate was 17.9% (95%CI, 17.6–18.1%) in a population of 27% women in Robson Group 1, 10.7% in Group 2, and 9.2% in Group 5.

**Conclusions:**

Multidisciplinary care following evidence-based protocols, associated with high motivation of both women and professionals for vaginal birth, may lead to a significant and safe reduction of CS rates even in contexts such as Brazil, with high medicalization of obstetric care and excess CS.

## Background

Cesarean section (CS) is a life-saving obstetric intervention for women and newborns in cases of complications during childbirth, but when performed without medical reasons, it may cause unnecessary short- and long-term complications. CS without clinical indications have increased globally, with exceptionally high levels in the private sector of large urban centers, motivating the World Health Organization (WHO) and government agencies to create strategies for its control [1, 2].

The proportion of births by CS per health service is a helpful indicator of obstetric care quality, and its audit is essential to understand trends and associated factors. Variations in the overall CS rate are challenging to interpret owing to heterogeneity in the infrastructure of health services, obstetric population, and protocols used [3, 4].

To facilitate the evaluation, comparison, and implementation of improvements, the WHO proposed the use of the Robson Classification, whose categories are fully inclusive and mutually exclusive, and which classifies women into homogeneous well-defined and clinically relevant groups based on obstetric variables at the time of admission to the labor ward [3, 4].

In 2019, Brazil ranked second globally with an overall CS rate of 57%, and the supplementary sector (health insurance sector) was responsible for financing 10% (287,166 of 2,849,146) of all births [5]. In this sector, the proportion of births by CS was 85% that year. According to the WHO, population based CS rates of 10–15% are associated with decreased maternal, neonatal, and infant mortality rates [4].

São Paulo is the Brazilian most populous city with a Human Development Index of 0.805 (very high according to the 2000 United Nations standards) and is in stage IV of the obstetric transition (low fertility and maternal mortality rate mainly due to indirect causes, being the strategy recommended for this stage to improve the qualification in care and the reduction of excess interventions in childbirth) and among the cities with the highest health insurance health coverage in Brazil (43%; 95% confidence interval [CI], 40–47%) [6, 7].

Appropriate interventions in childbirth, classified as clinical and nonclinical, may prevent unnecessary CS; however, clinical interventions can be harmful when overused [8, 9]. Clinical interventions guided by evidence-based protocols have a slight impact on CS rate because these protocols are not considered in countries with high CS rates such as Brazil. Examples include external cephalic version in cases of term breech presentation, vaginal breech birth in selected cases, vaginal birth after CS, neuraxial analgesia when indicated, labor induction in selected cases, and instrumental births for complications during the second stage [8]. On the other hand, nonclinical interventions tend to have a more significant ability to reduce CS rates because they influence the reduction of procedures without obstetric indications and include measures such as education about childbirth, continuous support in labor (provided by a companion of choice of the woman and by the presence of a doula), and care provided by midwives. Such interventions increase the rate of physiological births and lower the rates of complications, in addition to providing a positive experience of childbirth to women [8–10].

CS rates may vary according to facility complexity, epidemiological characteristics of the population treated, and care protocols used [2]. For this reason, the WHO C-model was developed to estimate the expected CS rate considering maternal age, obstetric characteristics used for Robson’s classification, and the incidence of comorbidities in the population, such as placenta previa, abruptio placentae, chronic hypertension, preeclampsia, kidney disease, HIV, and organic dysfunction with intensive care unit admission [2, 11].

Nordic countries (Denmark, Finland, Iceland, Norway, and Sweden) have maintained lower CS rates (approximately 17%) combined with good perinatal outcomes, and the region is considered a global reference for good obstetric practices. For this reason, Swedish data may be adopted as a gold standard. In these countries, childbirth care is less medicalized than in most high-income countries, low-risk births are managed by midwives, and obstetricians are called upon only when problems arise [2, 12].

This study presents CS rates by Robson’s group in a Brazilian population of women seeking a vaginal birth and a positive childbirth experience, demonstrating the possibility of obtaining CS rates closer to those calculated with the C-model tool by following evidence-based protocols and offering care and follow-up by a multidisciplinary team. In addition, it compares them with Sweden’s rates since, as mentioned, Nordic countries are considered a benchmark in perinatal care.

## Methods

This study adopted a cross-sectional design to evaluate CS rates by Robson group in women followed at a private practice (PP) in the city of São Paulo who gave birth in nine private hospitals in 2004–2019.

The inclusion criteria were women who sought prenatal care and childbirth with the intention of a spontaneous vaginal birth using appropriate technology, with single or multiple pregnancies, who gave birth in a hospital to a newborn or stillborn with a birth weight ≥ 500 g and/or gestational age ≥ 22 weeks. Women with planned home births were excluded, and there was no case of a woman undergoing CS at maternal request.

All women were followed up from prenatal care until birth by a multidisciplinary team led by a single obstetrician who was present at all births. The births were assisted in nine private hospitals according to the coverage of each woman’s health insurance. During the visits, the clinical and nonclinical aspects of childbirth care (perinatal education) were discussed to promote a positive experience that respected the women’s autonomy.

The same obstetrician was present at all births (including low-risk ones), and women were preferably admitted during the active phase (upon arrival, 3 contractions in 10 min with cervical effacement > 50% and dilation > 3 cm with intact or broken membranes). When a midwife was part of the team, the first evaluation was performed at home, with the obstetrician being called after 6 cm of cervical dilation was reached or earlier in case of intercurrence or the need for analgesia.

In labor, women had continuous support, presence of a companion and/or a doula of their choice, use of nonpharmacological methods for pain relief, freedom of movement during labor, free food ingestion, freedom of choice of position for birth, no use of a peripheral venous catheter, oxytocin or routine amniotomy, and completion of birth registered in their medical record [13].

The primary outcomes assessed were the overall CS rate, size of the Robson groups, CS rate in each group, and contribution to the overall CS rate from each group. The secondary outcomes were the frequency of clinical and nonclinical interventions in childbirth and sociodemographic characteristics (analyzed by a binary logistic regression model), the rate of vaginal birth, pre-labor CS, intrapartum CS, and the expected CS rate for the population using the WHO C-model tool [14, 15].

The databases were constructed and manipulated using Microsoft Excel and R Studio software (version 1.2.1335. 2009–2019) [16].

The database [17] and supplementary materials were shared in the Figshare open access repository (available at 10.6084/m9.figshare.17100314 and https://10.6084/m9.figshare.17057846 respectively).

The study was approved by the Research Ethics Committee of the Faculdade de Saúde Pública da Universidade de São Paulo through the National Plataforma Brasil under Certificate of Presentation of Ethical Appreciation number 50733621.8.0000.5421 on September 16, 2021 based on the Helsinki declaration. The ethical committee deemed that individual informed consent was not required given the fact that the dataset was made public and its information allowed confidential handling so that the data were presented without identifying individuals.

### Robson’s ten-group classification system

**Fig. 1:**
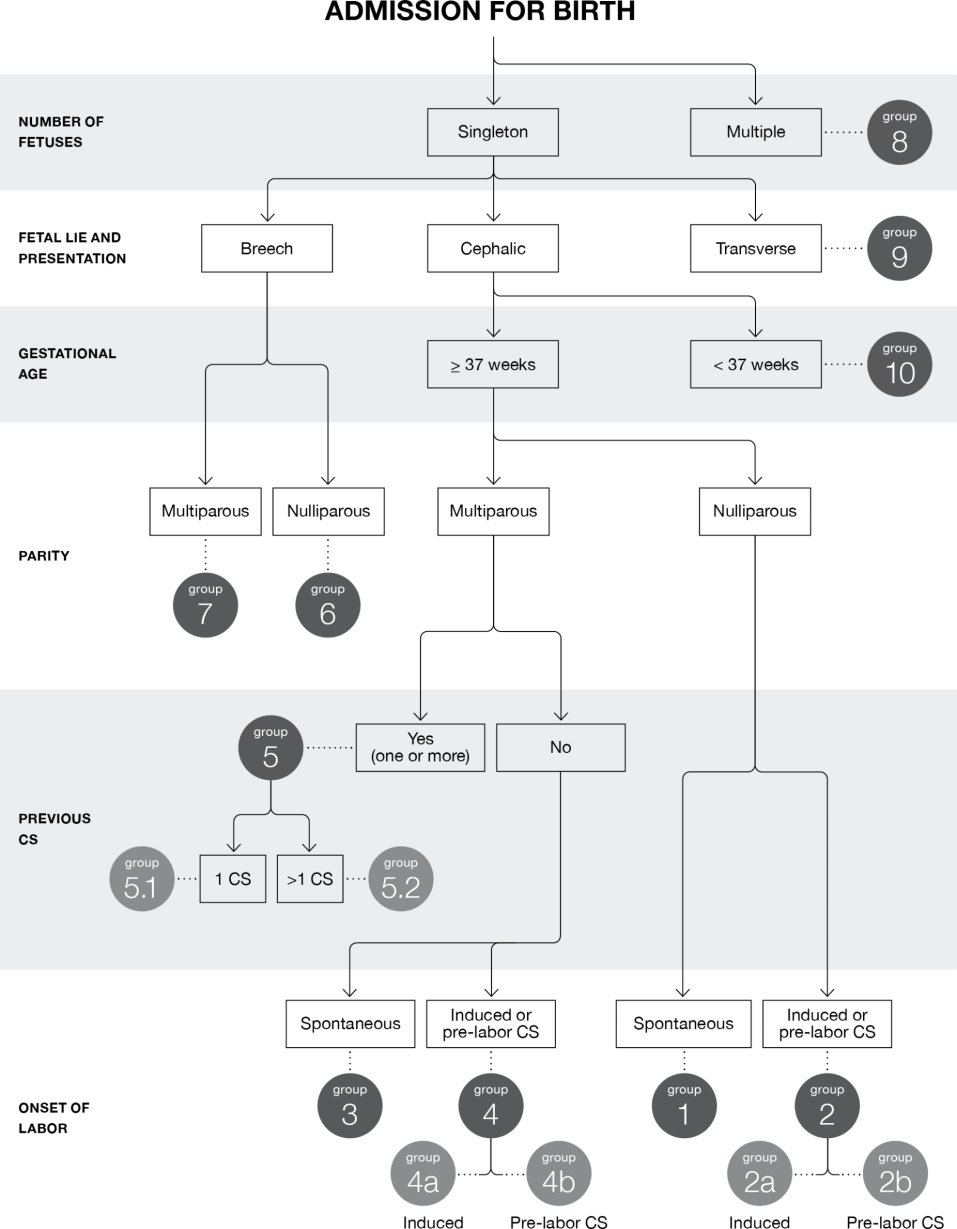
The Robson Classification with subdivisions Source: Authors’ elaboration based on World Health Organization data (2017) [2]. CS, cesarean section

Robson’s 10 groups classify women according to six obstetric variables at hospital admission for birth: the number of fetuses (single or multiple); fetal presentation (cephalic, breech, and transverse); parity (nulliparous or multiparous); gestational age (preterm or term); presence (or absence) of previous CS; and onset of labor (spontaneous, induced, and prelabor CS) (Fig. 1).

## Results

Table 1 describes the sociodemographic and obstetric characteristics of the 1,481 women who came from support groups for pregnant women and Internet search tools, sought this PP care, and chose to continue prenatal care at this PP service until birth in 2004–2019^15^.

**Table 1:** Distribution of women (n = 1481), cesarean sections (CS) and CS rates according to maternal characteristics in a private practice. Brazil, 2004–2019

	Women		CS		CS rate	
	No.	%	No.	%	%	P-value ^9^
*Age*						
< 20	1	0.1	0	0.0	0.0	**0.01**
20–34	946	63.9	123	54.9	13.0	
> 34	528	35.7	98	43.8	18.6	
Missing	6	0.4	3	1.3	50.0	
*Race* ^1^						
White	1333	90.0	194	86.6	14.6	0.60
Non-white	87	5.9	15	6.7	17.2	
Missing	61	4.1	15	6.7	24.6	
*Marital status (stable union)*						
With	1306	88.2	192	85.7	14.7	0.39
Without	159	10.7	28	12.5	17.6	
Missing	16	1.1	4	1.8	25.0	
*Higher Education*						
With	1344	90.7	201	89.7	15.0	0.20
Without	61	4.1	5	2.2	8.2	
Missing	76	5.1	18	8.0	23.7	
*Obstetric characteristics (Robson Group)*						
1 and 2 (NTSV) ^2^	815	55.0	124	55.4	15.2	**< 0.001**
3 and 4 (MTSVnoCS) ^3^	314	21.2	2	0.9	0.6	
5 (previous CS)	220	14.9	45	20.1	20.5	
6, 7 and 9 (NCP) ^4^	41	2.8	29	12.9	70.7	
8 (twins)	19	1.3	9	4.0	47.4	
10 (premature)	72	4.9	15	6.7	20.8	
High risk pregnancy ^5^	120	8.1	36	16.1	30.0	**< 0.001**
*Childbirth onset*						
Spontaneous labor ^6^	1163	78.5	115	51.3	9.9	**< 0.001**
Induced ^7^	264	17.8	55	24.6	20.8	
Pre-labor CS	54	3.6	54	24.1	100.0	
*Intrapartum care*						
Presence of Doula	928	62.7	132	58.9	14.2	0.24
Presence of Midwife	1090	73.6	133	59.4	12.2	**< 0.001**
Analgesia ^8^	623	42.1	170	75.9	27.3	**< 0.001**
**Total**	1481	100.0	224	100.0	15.1	

Source: PP data

Notes:

^1^according to provider

^2^nulliparous term singleton vertex

^3^multiparous term singleton vertex with no CS

^4^noncephalic presentation

^5^placenta previa, abruptio placentae, chronic hypertension, preeclampsia, kidney disease, HIV and organ dysfunction with intensive care unit admission

^6^on arrival, 3 contractions per 10 min with cervical effacement > 50% and dilation > 3 cm with intact or ruptured membranes

^7^use of misoprostol, Foley catheter, or oxytocin in a woman who does not fulfill the criteria for spontaneous labor

^8^pre-labor CS not included

^9^using Chi-squared test or Fisher’s exact test (maternal age)

CS, cesarean section; NCP, noncephalic presentation; MTSVnoCS, multiparous women at term with a single fetus in cephalic presentation without CS; NTSV, nulliparous women at term, with a single fetus in cephalic presentation (vertex)

For a clearer presentation, Robson’s groups were grouped according to their common obstetric characteristics: groups 1 and 2 represent nulliparous women at term, with a single fetus in cephalic presentation (vertex) (NTSV); groups 3 and 4 represent multiparous women at term with a single fetus in cephalic presentation without CS (MTSVnoCS); and groups 6, 7, and 9 represent those with noncephalic presentation (NCP).

Most women were 20–34 years of age, White, and with a stable union and higher education. Considering the obstetric characteristics, the largest group was NTSV, followed by MTSVnoCS and group 5 (multiparous, term, single fetus in cephalic presentation with CS). Altogether, these three groups were responsible for 91.1% of the population and 76.4% of CS births.

A doula and/or midwife were present in most cases (73,6% and 62.7%, respectively). During labor and birth, the women were encouraged to move around and use nonpharmacological methods for pain relief and free choice of position. Labor analgesia was administered at the women’s request in 623 births (42.1%).

There was a statistically significant positive association between CS and maternal age, high-risk pregnancy, childbirth onset, use of analgesia, and, of course, Robson group. A negative association was found in the presence of midwives.

The distribution of vaginal births, pre-labor CS, and intrapartum CS according to obstetric characteristics is displayed in Table 2, which shows a very low proportion of pre-labor CS. All CS were performed for clinical reasons (data not shown), and the proportion of pre-labor CS was low (3.6%) in the whole population and in all but the NCP group, which displayed a 61% pre-labor CS rate. Approximately 80% of women with a history of CS achieved a vaginal birth.

**Table 2:** Distribution of vaginal birth, pre-labor, and intrapartum CS of a private practice by Robson group, Brazil, 2004–2019

	Women		Vaginal birth		Pre-labour CS		Intrapartum CS	
Robson Group	N	%	N	%	N	%	N	%
1 and 2 (NTSV) ^1^	815	55.0	691	84.8	10	1.2	114	14.0
3 and 4 (MTSVnoCS) ^2^	314	21.2	312	99.4	0	0.0	2	0.6
5 (previous CS)	220	14.9	175	79.5	9	4.1	36	16.4
6, 7 and 9 (NCP) ^3^	41	2.8	12	29.3	25	61.0	4	9.8
8 (twins)	19	1.3	10	52.6	2	10.5	7	36.8
10 (premature)	72	4.9	57	79.2	8	11.1	7	9.7
**Total**	**1481**	**100.0**	**1257**	**84.9**	**54**	**3.6**	**170**	**11.5**

Source: PP data [17]

Notes:

^1^nulliparous term singleton vertex

^2^multiparous term singleton vertex with no CS

^3^noncephalic presentation

CS, cesarean section; NCP, noncephalic presentation; MTSVnoCS, multiparous women at term with a single fetus in cephalic presentation without CS; NTSV, nulliparous women at term with a single fetus in cephalic presentation (vertex)

Table 3 presents a Robson report table for PP and Sweden (S) as proposed by the WHO publication [2, 18]. There are important differences in group sizes. In PP, approximately 55% of women were nulliparous term singleton vertexes (groups 1 and 2), while in Sweden, these were approximately 40%. The multiparous term singleton vertex with no CS women (Groups 3 and 4) was around 20% in PP and around 40% in the Swedish population. Women with a previous history of CS (Group 5) represented 15% of the PP and 10% of the Swedish population. For both populations, the proportion of women in Groups 6–10 was < 10%. As for the CS rates, given the low proportion of women in Groups 3 and 4 in PP, no comparison was possible. The CS rates were similar for Groups 2a and 2b. For group 5, the PP CS rate was less than half of the Swedish rate (20% vs. 55%), with a PP success rate for vaginal birth after CS of approximately 80% and a vaginal birth after 2 CS of 70%. The PP CS rates were lower than the Swedish rates for Groups 6–10. The group that contributed the most to the overall CS rate (both absolute and relative) was Group 1 for PP versus Group 5 for Sweden.

**Table 3:** Robson report table for a private practice Brazil, 2004–2019 and Sweden, 2019

Group^I^	N CS / Total in group		Group size (%)^II^		Group CS rate (%) ^III^		Group contribution to overall CS rate (%)			
							Absolute^IV^		Relative^V^	
	PP	S	PP	S	PP	S	PP	S	PP	S
1	78/ 647	2259/ 30,356	43.69	27.02	12.06	7.44	5.27	2.01	34.82	11.26
2	46/ 168	4053/ 12,075	11.35	10.75	27.38	33.57	3.11	3.61	20.54	20.20
2a	36/ 158	2422/ 10,444	10.67	9.30	22.78	23.19	2.43	2.16	16.07	12.07
2b	10/ 10	1631/ 1631	0.68	1.45	100.00	100.00	0.68	1.45	4.46	8.13
3	1/ 272	577/ 37,718	18.37	33.58	0.37	1.53	0.07	0.51	0.45	2.88
4	1/ 42	1799/ 10,760	2.84	9.58	2.38	16.72	0.07	1.60	0.45	8.97
4a	1/ 42	399/ 9360	2.84	8.33	2.38	4.26	0.07	0.36	0.45	1.99
4b	0/ 0	1400/ 1400	0,00	1.25	0,00	100.00	0,00	1.25	0,00	6.98
5	45/ 220	5680/ 10,375	14.85	9.24	20.45	54.75	3.04	5.06	20.09	28.31
5.1	39/ 201	…	13.57	…	19.40	…	2.63	…	17.41	…
5.2	6/ 19	…	1.28	…	31.58	…	0.41	…	2.68	…
6	25/ 33	1794/ 1940	2.23	1.73	75.76	92.47	1.69	1.60	11.16	8.94
7	4/ 8	1024/ 1176	0.54	1.05	50.00	87.07	0.27	0.91	1.79	5.10
8	9/ 19	908/ 1640	1.28	1.46	47.37	55.37	0.61	0.81	4.02	4.53
9	0/ 0	355/ 375	0,00	0.33	0,00	94.67	0,00	0.32	0,00	1.77
10	15/ 72	1616/ 4903	4.86	4.36	20.83	32.96	1.01	1.44	6.7	8.05
**Total**	**224/ 1481**	**20,065/ 112,339**	**100.00**	**100.00**	**15.12**	**17.86**	**15.12**	**17.86**	**100.00**	**100.00**

Not classified: PP 0 cases, SP 1021 cases, 0.91%

Source: PP [17] data and SFOG [15]

Notes

I. Robson Groups:

1: Nulliparous women with a single cephalic pregnancy, ≥ 37 weeks gestation in spontaneous labor;

2: Nulliparous women with a single cephalic pregnancy, ≥ 37 weeks gestation who had labor induced (2a) or were delivered by CS before labor (2b);

3: Multiparous women without a previous CS, with a single cephalic pregnancy, ≥ 37 weeks gestation in spontaneous labor;

4: Multiparous women without a previous CS, with a single cephalic pregnancy, ≥ 37 weeks gestation who had labor induced (4a) or were delivered by CS before labor (4b);

5: All multiparous women with at least one previous CS, with a single cephalic pregnancy, ≥ 37 weeks gestation. 5.1: With one previous CS; 5.2: With two or more previous CSs;

6: All nulliparous women with a single breech pregnancy;

7: All multiparous women with a single breech pregnancy including women with previous CS(s);

8: All women with multiple pregnancies including women with previous CS(s);

9: All women with a single pregnancy with a transverse or oblique lie, including women with previous CS(s);

10: All women with a single cephalic pregnancy < 37 weeks gestation, including women with previous CS(s);

II. % = n of women in the group / total N women delivered in the setting x 100;

III. % = n of CS in the group / total N of women in the group x 100;

IV. % = n of CS in the group / total N of women delivered in the setting x 100;

V. % = n of CS in the group / total N of CS in the setting x 100;

… Data not available

CS, cesarean section; PP, private practice; S, Sweden

**Fig. 2:**
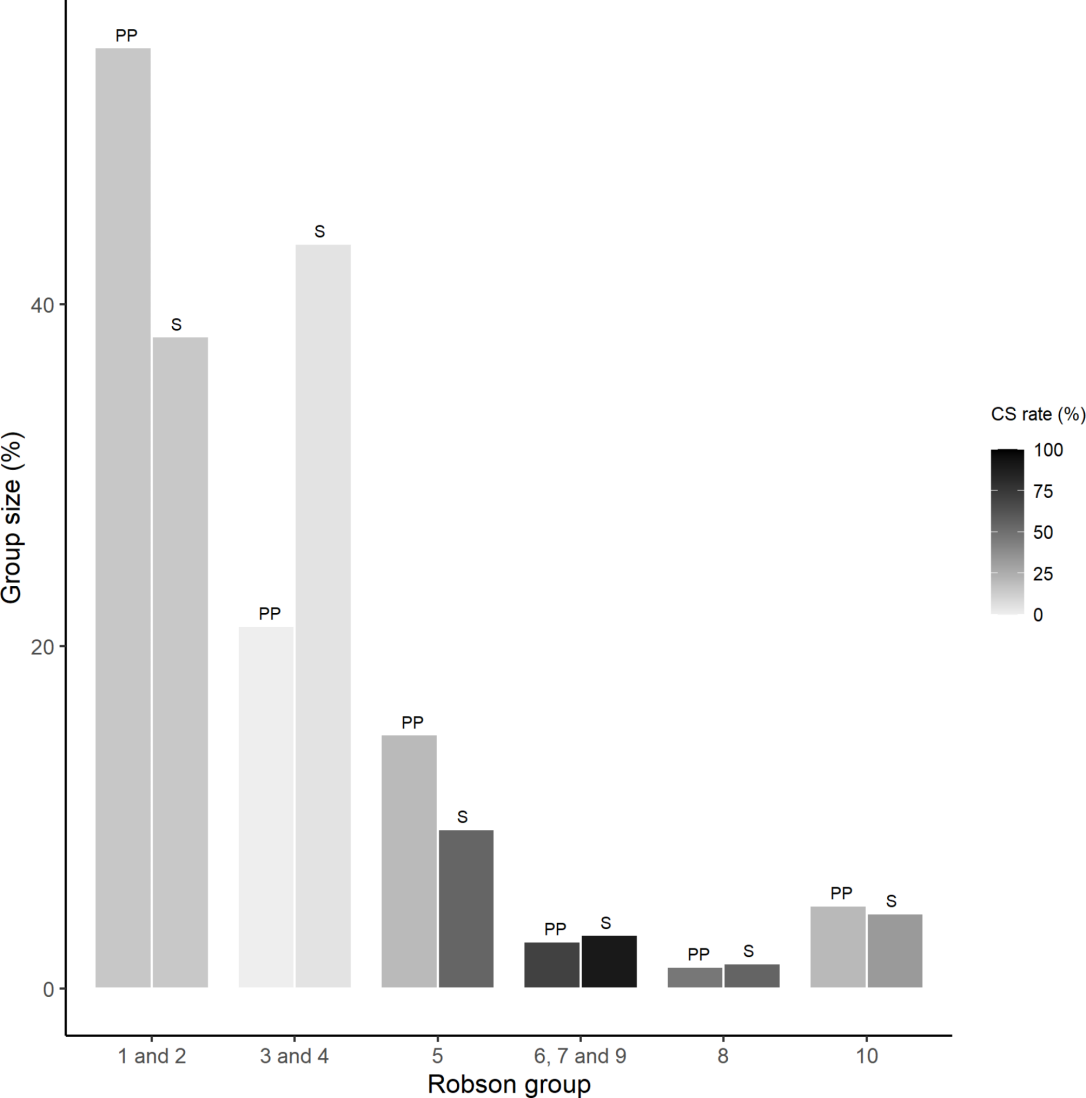
Robson group sizes and CS rates by common characteristics, private practice, Brazil, 2004–2019 and Sweden, 2019 Source: PP [17] data and SFOG [15] Notes: 1 and 2: nulliparous, term, singleton, vertex 3 and 4: multiparous, term, singleton, vertex, with no CS 5 previous CS 6, 7, and 9: noncephalic presentation 8: twins 10: preterm CS, cesarean section; PP, private practice; S, Sweden

Figure 2 displays the size and CS rates of the Robson groups by common characteristics for PP and Sweden. Group size reflects the proportion of women in each group, the darker the color, the higher the CS rate.

The submission of the PP database to the WHO c-model tool generated an expected CS rate for this population of 19.8% (95%CI 14.8 − 24.7%), with no statistically significant difference (considering the 95% CI) to the observed rate of 15.1% (CI 13.3 − 17.1%) [14].

## Discussion

The CS rate found in the PP was similar to that estimated by the C-model tool as well as to a database with low CS rates, Sweden [13], which displays excellent perinatal outcomes. For countries with high CS rates, such as Brazil, differences between rates observed and estimated by the C-model are usually higher [12, 19–21]. The groups that contributed the most to a lower rate in the PP were Groups 2 (particularly Group 2a) and 5, a result that also differs from the rates found in the WHO Global Survey of Maternal and Perinatal Health study [22].

Women of the PP population were mostly nulliparous. There was a low fertility rate since the PP presented a proportion of NTSV greater than that expected by the WHO. This group had low rates of induction of birth and pre-labor CS, reinforcing that such interventions were performed only for medical reasons [2, 23]. They were also able to pay for private services, and most (90.7%) had higher education, demonstrating that they were a population with a higher social level unlike Brazilian women in general, suggesting that social inequality in Brazil even affects obstetric care [24]. The proportion of preterm live births was similar to that of the Swedish population (approximately 5%).

Group 5 accounted for 14.9% of the population, and its size was related to the general CS rate. The larger group 5 size reflects a high population based CS rate, as it is represented by women nulliparous in their previous pregnancy who underwent CS. In places with low caesarean rates, it usually contributes to < 10% of women [2].

The CS rates of this PP were lower than those of other Brazilian studies published for all Robson groups considering that public and private sectors, including teaching hospitals, that should adopt the evidence-based protocols of the Brazilian Ministry of Health [25–28]. Studies that evaluated the quality of obstetric care practiced in the country evinced an excessively interventionist practice, such as the absence of evidence-based protocol use, with intervention rates even higher in high-income locations, no improvement in maternity service quality, and increases in risk of iatrogenic harm and cost [29–32].

According to the WHO, half of all CS occur in Group 5; the PP Group 5 displayed a CS rate of 20.5%, representing 20% of all CS performed. For Group 5, CS rates of 50–60% are expected [2, 23]. This rate contrasts even more when compared to a study in Brazilian teaching hospitals in 2021^26^. Of cases eligible for vaginal birth after CS, 95.3% had an elective CS and 39.2%, an intrapartum CS, suggesting that nonclinical factors lead to repeat CS in these services [26].

The significantly lower rates achieved in the PP may be due to the use of evidence-based protocols; women’s desire for a vaginal birth; professionals enabling all measures to facilitate it; continuous support for women during childbirth; for women in Group 5, the willingness of both the woman and the professionals to allow a trial of labor; absence of CS by maternal request because of self-selection; and the absence of obstetric indication for the previous CS typical of populations with high CS rates [33].

The collaborative and multidisciplinary model involving obstetricians and midwives is effective and successful for improving obstetric care (including high-risk cases), reducing adverse perinatal events, and increasing safety and women’s satisfaction with their birth experience; it encourages a patient- and family-centered practice and is tuned to the WHO recommendations for a positive childbirth experience. Studies that compared the prevalence of CS and neonatal outcomes in two models of childbirth care, including a study in a Brazilian private hospital, showed a lower CS rate with the model of care offered by a multidisciplinary team and no difference in neonatal outcomes [32, 34–37].

Another possible explanation for the higher CS rates in other private practices relates to the fact that many Brazilian gynecologists and obstetricians perform defensive CS, usually due to fear of litigation. Vaginal birth can be perceived as unpredictable and with a higher risk of litigation by health care professionals; therefore, they opt for defensive CS [38]. Women’s desire for a vaginal birth and their access to quality information reinforce their autonomy for informed choices and thus may contribute to a better childbirth experience and decreased risk of litigation in cases of complications.

The strengths of this study are that all cases were assisted by the same team that was concerned about meticulous registration of procedures, and the quality of the data is supported by the fact that no woman was omitted from the Robson’s group classification.

Potential limitations include: its population was composed primarily of women seeking a vaginal birth, while those at higher risk for CS or with a contraindication for a vaginal birth might have been excluded; all women were from the private sector which, in this town, represents less than half of the population; all patients were cared for by a team with the same leader and, although the data were collected throughout 15 years, changes in the team and procedures were not considered, which could be a major confounder. Maternal and neonatal outcomes were not the object of this study. Another limiting factor is the low coverage of midwives given the additional fee charged by them, which might have discouraged women who had their follow-up during antenatal care to hire them for their birth. The low coverage of such midwifery services is a barrier to the implementation of collaborative care in the US [39].

The results of this study demonstrate the real possibility of achieving CS rates according to those displayed in WHO publications by offering multidisciplinary childbirth care that follows evidence-based protocols and proposes to provide a positive childbirth experience as recommended by the WHO. Health policies should promote health education for the population and health professionals that focus on disseminating and implementing such practices in the public and private sectors.

## Conclusion

Multidisciplinary care following evidence-based protocols and a high motivation of both women and professionals of childbirth care for a vaginal birth may lead to a significant reduction in CS rates even in contexts such as Brazil, where there is high medicalization of obstetric care and an excess CS rate.

Such professional and women’s motivation may be improved by government educational campaigns that would promote childbirth care towards a positive childbirth experience as recommended by the WHO as well as by the implementation of the collaborative care model with midwives in the public and private sectors.

## Data Availability

The datasets generated and/or analysed during the current study are available in the Figshare repository at 10.6084/m9.figshare.17100314 and 10.6084/m9.figshare.17057846 (R script).

## List of Abbreviations

CI: Confidence interval
CS: Cesarean section
MTSVnoCS: Multiparous women at term with a single fetus in cephalic presentation without CS
NCV: Noncephalic presentation
NTSV: Nulliparous women at term with a single fetus in cephalic presentation (vertex)
PP: Private practice
WHO: World Health Organization
